# Evidence for NG2-glia Derived, Adult-Born Functional Neurons in the Hypothalamus

**DOI:** 10.1371/journal.pone.0078236

**Published:** 2013-10-29

**Authors:** Sarah C. Robins, Eric Trudel, Olivia Rotondi, Xiaohong Liu, Tina Djogo, Darya Kryzskaya, Charles W. Bourque, Maia V. Kokoeva

**Affiliations:** 1 Department of Medicine, McGill University, Montreal, Canada; 2 Centre for Research in Neuroscience, McGill University, Montreal, Canada; Institut National de la Recherche Agronomique-CNRS UMR6175, France

## Abstract

Accumulating evidence suggests that the adult murine hypothalamus, a control site of several fundamental homeostatic processes, has neurogenic capacity. Correspondingly, the adult hypothalamus exhibits considerable cell proliferation that is ongoing even in the absence of external stimuli, and some of the newborn cells have been shown to mature into cells that express neuronal fate markers. However, the identity and characteristics of proliferating cells within the hypothalamic parenchyma have yet to be thoroughly investigated. Here we show that a subset of NG2-glia distributed throughout the mediobasal hypothalamus are proliferative and express the stem cell marker Sox2. We tracked the constitutive differentiation of hypothalamic NG2-glia by employing genetic fate mapping based on inducible Cre recombinase expression under the control of the NG2 promoter, demonstrating that adult hypothalamic NG2-glia give rise to substantial numbers of APC+ oligodendrocytes and a smaller population of HuC/D+ or NeuN+ neurons. Labelling with the cell proliferation marker BrdU confirmed that some NG2-derived neurons have proliferated shortly before differentiation. Furthermore, patch-clamp electrophysiology revealed that some NG2-derived cells display an immature neuronal phenotype and appear to receive synaptic input indicative of their electrical integration in local hypothalamic circuits. Together, our studies show that hypothalamic NG2-glia are able to take on neuronal fates and mature into functional neurons, indicating that NG2-glia contribute to the neurogenic capacity of the adult hypothalamus.

## Introduction

Several studies published over the past years suggest that the adult hypothalamus has a neurogenic capacity [1]–[7]. In particular, adult-born cells expressing neuronal fate markers have been found in the adult mediobasal hypothalamus. This region plays a key role in energy balance regulation, and there is evidence that manipulating hypothalamic cell proliferation affects body weight and food intake [1], [5]–[7]. Research into the origins of this neural genesis has recently identified third ventricular tanycytes as a neurosphere-forming cell type, capable of producing multiple neural lineages in vitro [3], [6], [8], [9]. Tanycytes have also been demonstrated in vivo to mature into cells that express neuronal markers during postnatal development [5] and in the adult [9], [10]. It is plausible that tanycytes represent the major source for hypothalamic cells that take on neuronal fates, however the number of tanycyte-derived neurons as assessed by genetic fate mapping appears to be low when compared to the total number of newborn hypothalamic neurons observed by bromo-desoxyuridine (BrdU) incorporation [2], [9]. Because hypothalamic cell proliferation is not restricted to the vicinity of the third ventricle [2], it is conceivable that local parenchymal precursors may additionally contribute to the hypothalamic neurogenic potency. While several studies reported that the adult hypothalamus gives rise to cells that express neuronal markers such as NeuN or HuC/D (Hu), electrophysiological evidence for a neuronal identity of adult born hypothalamic is still lacking.

NG2-glia are one of the few cell types that continue to divide in the adult brain [11], [12]. While they are well known for their role as precursors for myelin-forming oligodendrocytes [13]–[15], knowledge about their functional significance in the adult brain, particularly in the grey matter, is still rudimentary [14], [16], [17]. NG2-glia account for up to 10% of cells in the adult CNS and are defined by their expression of nerve-glia antigen 2 (NG2), a chondroitin sulfate proteoglycan [18], [19], and platelet derived growth factor receptor alpha (PDGFRα) [20]. Of note, it has been suggested that NG2-glia can give rise to a limited number of neurons in the adult piriform cortex [13], [21], [22], although other studies have challenged this view [14], [15], [23]. Rivers et al [13] employed genetic fate tracing to reveal that Pdgfrα expressing cells give rise to some projection neurons in the piriform cortex of adult mice. This was later confirmed using a different Cre recombinase driver, the promoter for proteolipid protein (Plp), which represents another specific NG2-glia marker protein [21]. However, others using Olig2-CreER [14] or PDGFRα-CreER mice [23], [24] could not confirm these results or argued that the new piriform neurons are not born from locally residing progenitors [23]. The findings prompted us to explore whether hypothalamic NG2 cells could act as parenchymal stem cells, accounting for some of the neuronal fate marker positive, newborn hypothalamic cells that we and others previously observed. Here, we show that hypothalamic NG2-glia proliferate and self-renew, and that daughter cells of dividing NG2-glia differentially express Sox2. We further provide evidence by NG2-CreER-based fate tracing that dividing hypothalamic NG2-glia mature into cells possessing the electrophysiological properties of neurons. These findings are in support of a stem cell role of hypothalamic NG2 glia and corroborate that the adult hypothalamus exhibits a neurogenic capacity.

## Results

### Mitotically Active Cells are Distributed Throughout the Adult Hypothalamic Parenchyma and Predominantly Express NG2

**Figure 1:**
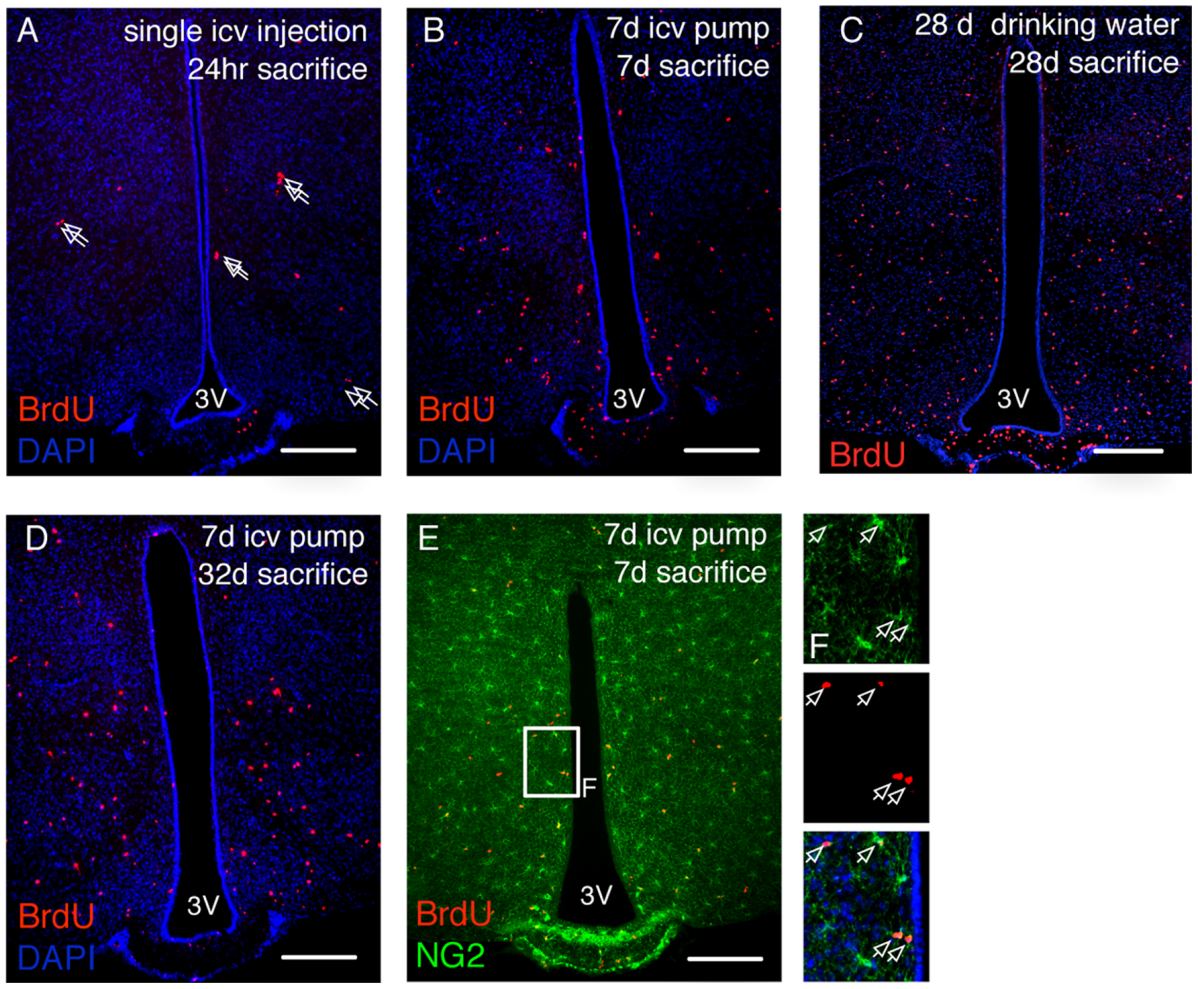
Distribution of newborn cells in the adult hypothalamus. BrdU incorporation into hypothalamic cells as a measure of cell proliferation was assessed after a single icv injection of BrdU (**A**), a 7 day icv infusion (**B**), or a 28 day exposure to BrdU-containing drinking water (**C**). Arrows in **A** indicate BrdU+ doublets. (**D**), distribution of hypothalamic BrdU+ cells at day 32 after the initiation of a 7 day icv BrdU infusion. (**E**), labelling for both BrdU (red) and NG2 (green) reveals numerous BrdU positive NG2-glia cells. Boxed area is shown enlarged to the right, arrows mark BrdU+ cells. Scale bars = 200 µm.

In order to identify the source of adult-born hypothalamic cells, we cerebroventricularly infused mice with BrdU to label dividing cells. BrdU is a proliferation marker that is incorporated into the replicating DNA during S-phase of the cell cycle. One day after BrdU delivery into the ventricular system, inspection of coronal brain sections at the level of the mediobasal hypothalamus revealed BrdU positive cells in the hypothalamic parenchyma. These cells were not confined to the vicinity of the third ventricle, but were mostly found in the more distal parenchyma (Fig. 1A). BrdU+ cells were frequently observed in closely apposed pairs (Fig. 1A, arrows) deep inside the parenchyma, arguing that these cells have divided locally and did not migrate long distances subsequent to cell division. The general pattern of BrdU+ cell distribution did not change after infusion for seven days or after BrdU had been provided for 28 days in the drinking water (Fig. 1B,C): BrdU+ cells were found spread throughout the hypothalamic parenchyma without any obvious regional enrichment. This was also true for BrdU+ cells occurring in pairs, corroborating that cell birth in the adult hypothalamus is not spatially restricted. A pulse-chase experiment in which mice were BrdU-infused for seven days and then sacrificed at 28 days post-surgery yielded a parenchymal distribution of BrdU+ cells (Fig. 1D) similar to that observed directly after the cessation of BrdU infusion (seven days post surgery, Fig. 1B). This finding is further support for the notion that adult-generated hypothalamic cells do not substantially migrate after their birth. Given that NG2-glia have been demonstrated to continuously proliferate in the adult brain [11], [12], we assessed whether the BrdU+ cells we detected in the hypothalamus express NG2. Co-labelling with an NG2 antibody that is specific to NG2-glia revealed that the majority of BrdU-positive cells are NG2+ (Fig. 1E), with BrdU+/NG2+ cells showing no regional restriction. When brains are inspected 24 hours after a single BrdU injection, approximately 80% of BrdU+ cells are found to express NG2, a proportion that decreased over time, likely due to differentiation into other cell types that no longer express NG2 [25]. Together, these results indicate that new cells are born throughout the adult hypothalamic parenchyma, and that NG2-glia contribute substantially to the dividing cell population.

### Stem Cell Marker Expression in Hypothalamic NG2-glia

**Figure 2:**
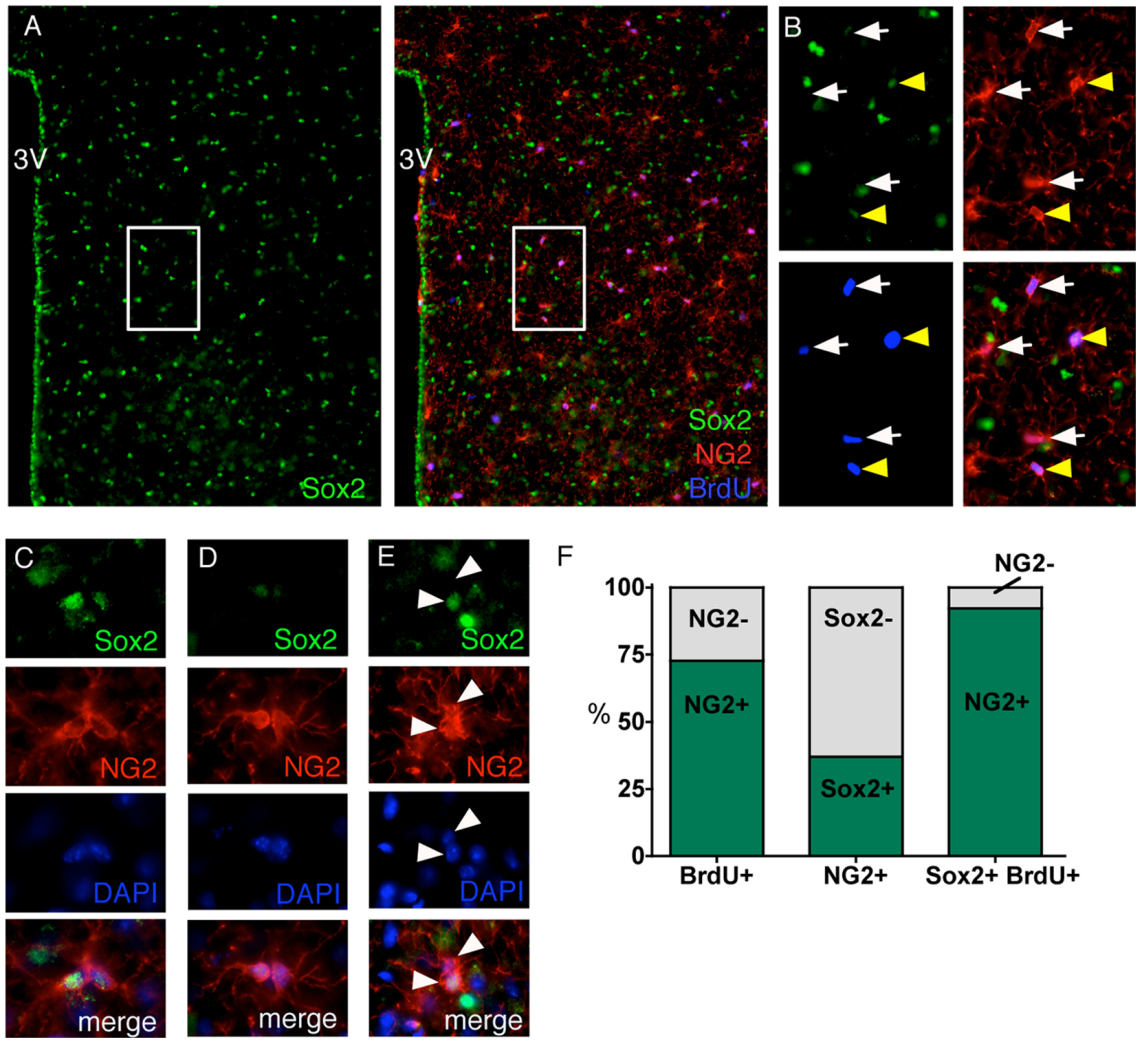
Stem cell features of NG2-glia. Mice were treated with BrdU in the drinking water for 28 days before sacrifice to mark proliferating cells. Sox2 (green), NG2 (red) and BrdU (blue) mark overlapping populations of cells (**A**, box enlarged in **B**). NG2+/BrdU+ cells were found either Sox2+ (yellow arrowheads in **B**) or showed no or barely detectable immuno-signal for Sox2 (denoted as Sox2-, white arrows in **B**). As NG2-glia are usually found in a well-spaced tiled pattern, pairs of adjacent NG2-glia are likely to represent recently-divided cells. In NG2 doublets, Sox2 was appreciable (**C**), absent or barely detectable (**D**), or limited to one cell (**E**; white arrowheads indicate nascent NG2-glial cell pair). (**F**), Quantification of the proportion of BrdU+ cells that also expressed NG2 (bar, left), the number of NG2-glia cells that also expressed Sox2 (bar, middle), and the number of Sox2+/BrdU+ cells that also expressed NG2 (bar, right); n = 3 mice.

In addition to their undisputed role as oligodendrocyte progenitors, NG2-glia have been associated with the occurrence of new neurons specifically in the piriform cortex, suggesting a potential stem cell role of these glia. Of interest in this context is a recent study that explored the fate of hypothalamic Sox2 expressing cells by viral in vivo delivery of a Sox2-Cre transgene into the hypothalamic region of an adult YFP reporter mouse [7]. The authors found YFP+ cells that expressed neuronal markers 80 days post viral injection, indicating that at least some adult hypothalamic Sox2+ cells are neurogenic. Sox2 is known for its pluripotency maintenance role in embryonic stem cells [26], [27] and has been detected in both subventricular zone B-cells and dentate gyrus progenitors [28], [29] suggesting that this transcription factor has a role in adult neural stem cell function as well. These results prompted us to investigate the expression of NG2 and Sox2 in the adult hypothalamus by immunofluorescent co-labelling. In order to assess the proliferative status of NG2+ and Sox2+ cells, we provided BrdU through the drinking water for 28 days before perfusion. We found Sox2 to be expressed in the third ventricle lining and in many cells evenly distributed throughout the hypothalamic parenchyma (Fig. 2A). The immunological signal for Sox2 was exclusively nuclear, as expected for a transcription factor. Unlike the immune signal for NG2 which did not vary much with regard to its strength, we observed Sox2 fluorescence to vary substantially within the hypothalamus, with some cells exhibiting strong staining while others showed only weak signals. To minimize the risk of false positive sampling, only strong unambiguous Sox2 labelling was quantified. Co-labelling with NG2 and Sox2 antibodies indicated that 37.0% ±2.8 of hypothalamic NG2-glia robustly express Sox2 (Fig. 2A,B,F). These Sox2-expressing NG2-glia were distributed throughout the hypothalamic parenchyma without any obvious areas of enrichment. Examination of the BrdU+ cells in this experiment revealed that the majority of them expressed NG2, as expected (72.7±6.0%, Fig. 2A,B,F). Despite the fact that NG2-glia accounted for fewer than 10% of the total Sox2+ cell population, almost all (92.2%±2.8) of the hypothalamic BrdU+/Sox2+ cells also expressed NG2 (Fig. 2A,B,F), suggesting that NG2-glia are the only substantially proliferative subpopulation of Sox2+ cells in the adult hypothalamic parenchyma. Interestingly, when we examined doublets of NG2-positive cells, i.e. NG2 cells that were closely apposed and thus likely resulted from a very recent cell division, we found three types of NG2-glia pairs according to Sox2 expression: i) substantial Sox2 expression in both cells; ii) no to barely detectable Sox2 expression in both cells; or iii) Sox2 expression limited to one cell (Fig. 2C–E). These differences in Sox2 expression may relate to differences in neural fates. BrdU positive NG2+ cells not associated with mitotic figures/cell pairs varied with respect to their Sox2 expression status (Fig. 2B), which may reflect different maturation states, distinguish different NG2-glia cell fates, or define different sub-classes of hypothalamic NG2-glia.

### Self-renewal of Hypothalamic NG2-glia

**Figure 3:**
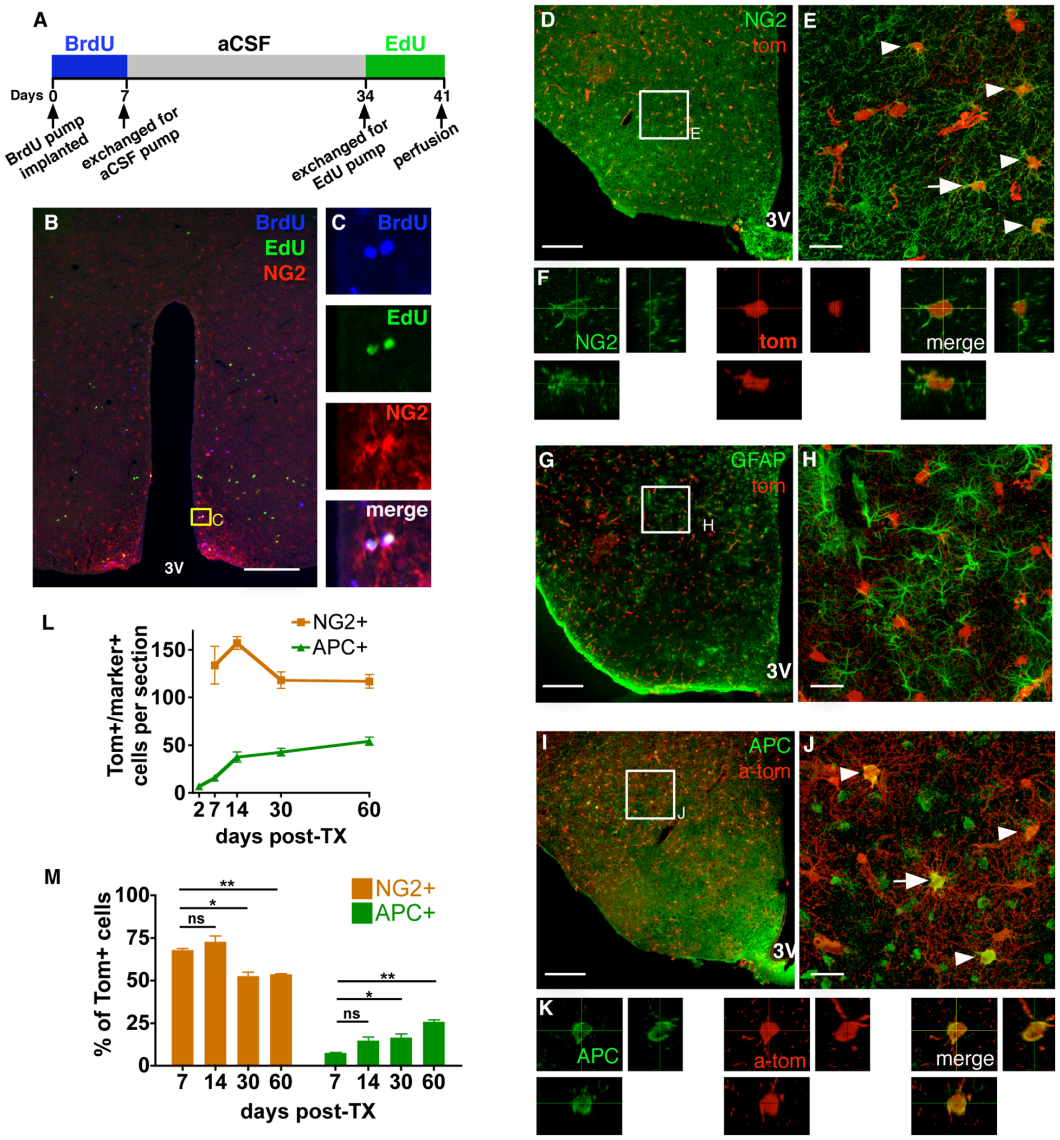
NG2-glia self-renewal and differentiation to glial lineages. (**A**), Schematic of the regimen for sequential labelling with the mitotic markers BrdU and EdU. (**B,C**), Immunohistochemical analysis after sequential BrdU and EdU treatment demonstrates self-renewal of NG2+ cells. (**D–M**) Fate tracing of NG2-glia in the adult hypothalamus of NG2CreER:tdTomato mice. Animals were treated with TX, and sacrificed 2, 7, 14, 30 or 60 days after the first injection. Markers for NG2-glia (NG2; **D–F**), astrocytes (GFAP; **G–H**) and oligodendrocytes (APC; **I–K**) were examined for co-labelling in Tom+ cells. All images are from the 30 day timepoint, arrows and arrowheads indicate co-labelled cells. (**F,K**), 3D reconstructions from a z-stack of confocal images of the cell marked by an arrow in **E** and **J**. Tom+/APC+ and Tom+/NG2+ cells were quantified over 60 days post-TX, both as the number of cells per section (**L**) and as a proportion of the total Tom+ cells (**M**). **E, H, J,** maximum intensity projections of confocal Z-stacks throughout section thickness. All graphs show mean ± SEM, n = 3; scale bars = 20 µm (**E,H,J**) or 200 µm (**D,G,I**). Student’s T-test, *p<0.05, **p<0.005, ns = not significant.

To address the capacity of hypothalamic NG2-glia to continuously self-renew, a stem cell hallmark, we performed sequential intracerebroventricular (icv) infusion of the mitotic markers BrdU and ethynyl-deoxyuridine (EdU) 28 days apart (see schematic in Fig. 3A). More than two thirds of the detected BrdU+/EdU+ cells expressed NG2 (Fig. 3B,C), indicating that adult hypothalamic NG2-glia can undergo multiple self-renewing divisions, in line with findings for the cerebral cortex [11]. The observation that most of the hypothalamic BrdU+/EdU+ cells are NG2+ further suggest that NG2-glia are the predominant hypothalamic cell type to undergo self-renewing divisions.

### Tracking NG2-glia Fates Using NG2creER:tdTomato Mice

Given that NG2-glia in the adult hypothalamus self-renew and that a distinct fraction of mitotically active NG2-glia express the pluripotency marker Sox2, we speculated that hypothalamic NG2-glia may mature into postmitotic cells of different fates. To assess differentiation of hypothalamic NG2-glia we crossed NG2creER mice [30] with a tdTomato (Tom) reporter line which expresses the red fluorescent protein Tom upon removal of a *LoxP*-flanked (floxed) transcriptional stop cassette through the recombinatorial action of Cre recombinase. In the resulting NG2CreER:tdTomato line, the stop cassette is permanently removed from NG2+ cells in response to tamoxifen (TX) treatment, and thus the affected NG2+ cells and their offspring will be marked by Tom expression. As previously reported, we see a high rate of recombination in NG2-glia, with 80.2% ±2.5 of NG2+ cells also expressing Tom [25]. This level of recombination is reached within seven days following the first TX injection (two days after the final injection), beyond which time it remains stable. Validation of the fidelity of the Cre transgene was undertaken at an early timepoint in order to minimise the likelihood of including differentiated cells. Given that the number of APC+ oligodendrocytes increased sharply between 48 hours and seven days post-injection (see Fig. 3L), we concluded that substantial differentiation can occur within five days. We therefore used mice sacrificed 48 hours after the first TX injection, at which point the recombination level had reached about four-fifths of its final value. The majority of Tom+ cells are identified by antibody labelling as NG2-glia (75.6% ±0.8, Fig. S1A,B). As predicted for this early time point after TX treatment onset, only a few Tom+ cells showed a more differentiated phenotype reflected by the co-expression of the APC oligodendrocyte marker and NG2 (1.4%) or expression of APC alone (3.0% ±0.0). The remainder of the Tom+ cells expressed the pericyte marker CD13 (21.3% ±0.9). Pericytes are smooth muscle vascular cells which are known to express NG2 [31], although they are not recognized by the NG2 antibody utilised here. Notably, BrdU labelling was never observed in pericytes, nor were they susceptible to ablation using mitotic inhibition [25] indicating that pericytes are post-mitotic in the adult hypothalamus and likely elsewhere in the brain. The three antibodies utilised here, detecting NG2, APC, or CD13, were sufficient to identify all cells of the Tom+ population. Accordingly, Tom fluorescence was never colocalised with the immune signal for the neuronal markers HuC/D or NeuN at this timepoint (Fig. S1C,D). Thus, Tom is faithfully expressed in the expected populations of NG2+ cells at this early time point post TX treatment initiation.

### Oligodendroglial Differentiation of Hypothalamic NG2-glia

To examine the cell fate of NG2-glia over time, mice were sacrificed at different time points, up to 60 days after TX treatment. At 30 days post-TX, we observed that many Tom+ cells still expressed NG2 (Fig. 3D–F). In fact, the absolute number of Tom+ NG2-glia stayed roughly constant over time (7 days vs. 60 days p = 0.40, Fig. 3L), suggesting that they are lost to cell death or differentiation at approximately the same rate as they are generated by self-renewing divisions. At all time points, Tom+ cells were negative for the astrocyte marker GFAP (Fig. 3G,H), which implies that adult hypothalamic NG2-glia do not differentiate into astrocytes, confirming earlier findings in both grey and white matter [13].

We next assessed differentiation along the oligodendrocyte lineage, using APC as a marker. As already pointed out above (Fig. S1), a few Tom+/APC+/NG2± cells could be already detected at the earliest (2 days) time point after initiation of TX treatment. The number of hypothalamic Tom+/APC+ cells increased over time (Fig. 3I–M), and by 60 days post-induction 25.6% ±1.2 of Tom+ cells were oligodendrocytes. In order to assess the degree of oligodendrogenesis after the cessation of recombination, the proportion of APC+/Tom+ cells was compared between seven days post-induction (mice sacrificed two days after the cessation of the five day TX regime, beyond which time we saw no further recombination in NG2-glia) and later timepoints. The proportion of oligodendrocytes was substantially increased by 60 days post induction whereas the NG2+ fraction of Tom+ cells decreased during the same time period (Fig. 3M). This is in agreement with findings in other adult CNS regions [13], [14], [24], corroborating that one of the primary functions of adult hypothalamic NG2-glia is to provide a continuous supply of new oligodendrocytes.

### Neuronal Differentiation of NG2-glia

Some of the previous research on NG2-glia fates in the adult brain showed that NG2-glia are not limited to function as unipotent oligodendrocyte precursors, but can also produce new neurons, most notably in the piriform cortex [13], [21], [22].

**Figure 4:**
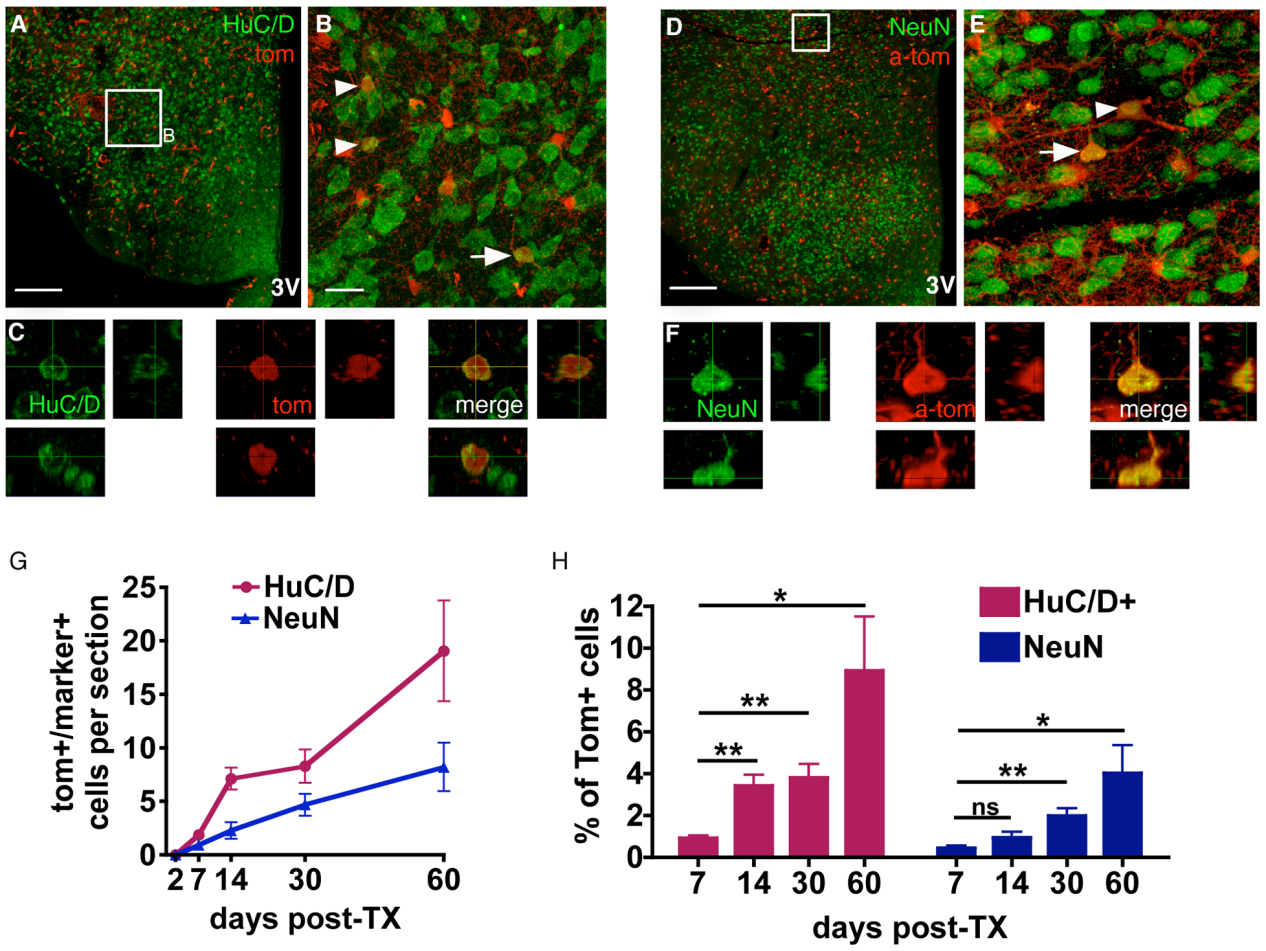
NG2-glia differentiation to the neuronal lineage. Confocal images of sections from mice perfused 30 days post-TX were stained with antibodies against the neuronal markers Hu (**A–C**) and NeuN (**D–F**). In (**D–F**) Tom signal is enhanced by co-labelling with an anti-Tomato (a-tom) antibody. Arrows and arrowheads mark colabelled cells. Cells marked by arrows are reconstructed in 3D from a z-stack of confocal images (**C,F**). (**G,H**) Quantification of Tom+/NeuN+ and Tom+/Hu+ cells at 2, 7, 14, 30 and 60 days after initiation of TX treatment, shown as absolute numbers (**G**), and as a proportion of all Tom+ cells (**H**). **B, E** maximum intensity projections of confocal Z-stacks. All graphs show mean ± SEM; n = 6; scale bars = 20 µm (**B,E**) or 200 µm (**A,D**). Student’s T-test, *p<0.05, **p<0.005, ns = not significant.

In order to assess neuronal identity we employed antibodies against Hu, which represents a lineage restricted marker for immature and mature neurons [32], [33] (Fig. 4A–C), and antibodies against NeuN, which is found in the majority of mature neuronal cells [34] (Fig. 3D–F). An immunochemical signal for these neuronal markers was never detected in Tom+ cells at the earliest inspection time point, two days after TX treatment onset (Fig. S1C,D, Fig. 4G), with the first neuronal marker-positive Tom+ cells appearing between two days and seven days post-induction. A successive emergence of colabelled cells was observed over 60 days following induction (Fig. 4G), by which time 8.6% ±2.6 of Tom+ cells expressed Hu and 4.0% ±1.3 expressed NeuN (Fig. 4H). The proportion of Tom+ cells expressing either neuronal marker appeared to accumulate in a linear manner, with a doubling of the time since induction resulting in a doubling of the number of neurons observed. This is indicative of a constant rate of neuron production.

When adjusted for the Cre recombination rate (80.2% ±2.5 [25]), we calculated that 0.90% of all Hu+ neurons in the adult hypothalamus had been derived from NG2-glia within the time period of 60 days. For NeuN+ neurons this value was 0.54%. This difference may be partially accounted for by the more ubiquitous expression of Hu in the hypothalamus: Hu+ cells were overall 1.3 times more abundant than NeuN+ cells [2269 vs. 1745 per section respectively], with NeuN being largely undetectable in the arcuate nucleus, and indistinct in the dorsomedial hypothalamus. The predominance of Tom+/Hu+ vs. Tom+/NeuN+ cells may also reflect a prevailing immature status among newly differentiated NG2-glia derived neurons, or a bias towards differentiation into Hu+/NeuN- populations.

**Figure 5:**
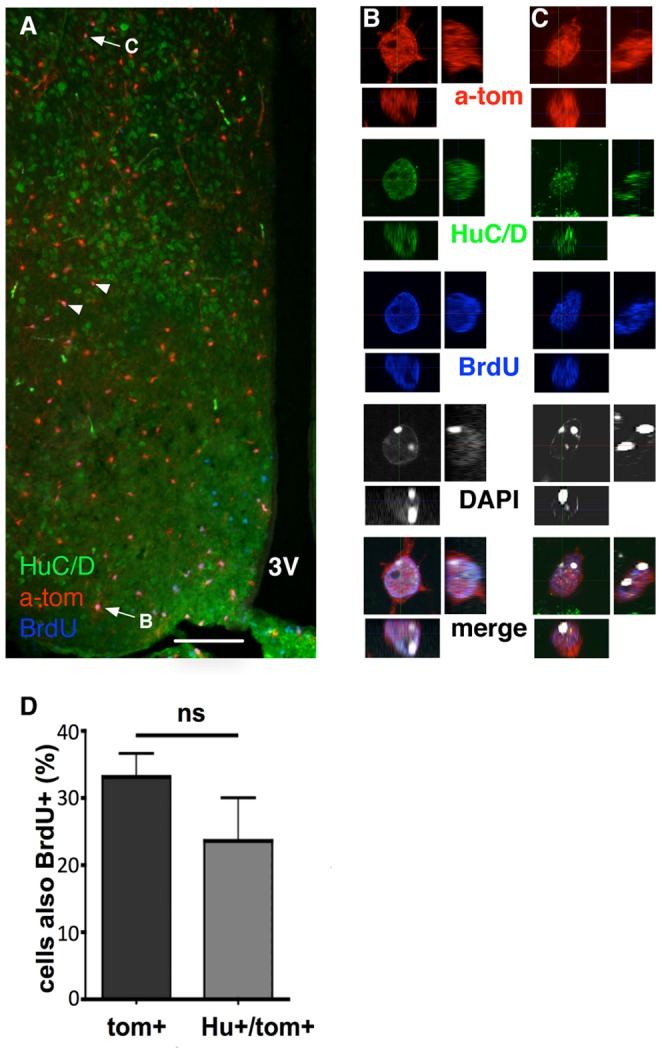
BrdU incorporation in NG2-derived neurons. BrdU was continuously provided in drinking water from the first day of TX injections until sacrifice 28 days later. Hu+/Tom+/BrdU+ triple positive cells were identified in the hypothalamus (arrows/arrowheads in **A**), and confirmed by 3D reconstruction from confocal Z-stacks (**B,C**; marked by arrows in **A**). There was no significant difference (p>0.05) between the proportion of Tom+ cells that also contained BrdU, and the proportion of Tom+/Hu+ cells that also contained BrdU (**D**). Scale bar = 200 µm.

When BrdU was provided for 28 days continuously in the drinking water, starting on the first day of TX treatment, we detected Hu+/Tom+ cells that were also positive for BrdU (Fig. 5), indicating that they differentiated from NG2-glia that had divided during the period of BrdU delivery. Notably, the percentage of BrdU+/Hu+/Tom+ from total Hu+/Tom+ cells was not significantly different from the percentage of BrdU+/Tom+ cells from total Tom+ cells (Fig. 5D), suggesting that the number of BrdU+/Hu+/Tom+ cells detected depends on the proportion of NG2-glia prelabelled with BrdU.

### NG2-glia Derived Cells Exhibit Electrical Properties of Neurons

**Figure 6:**
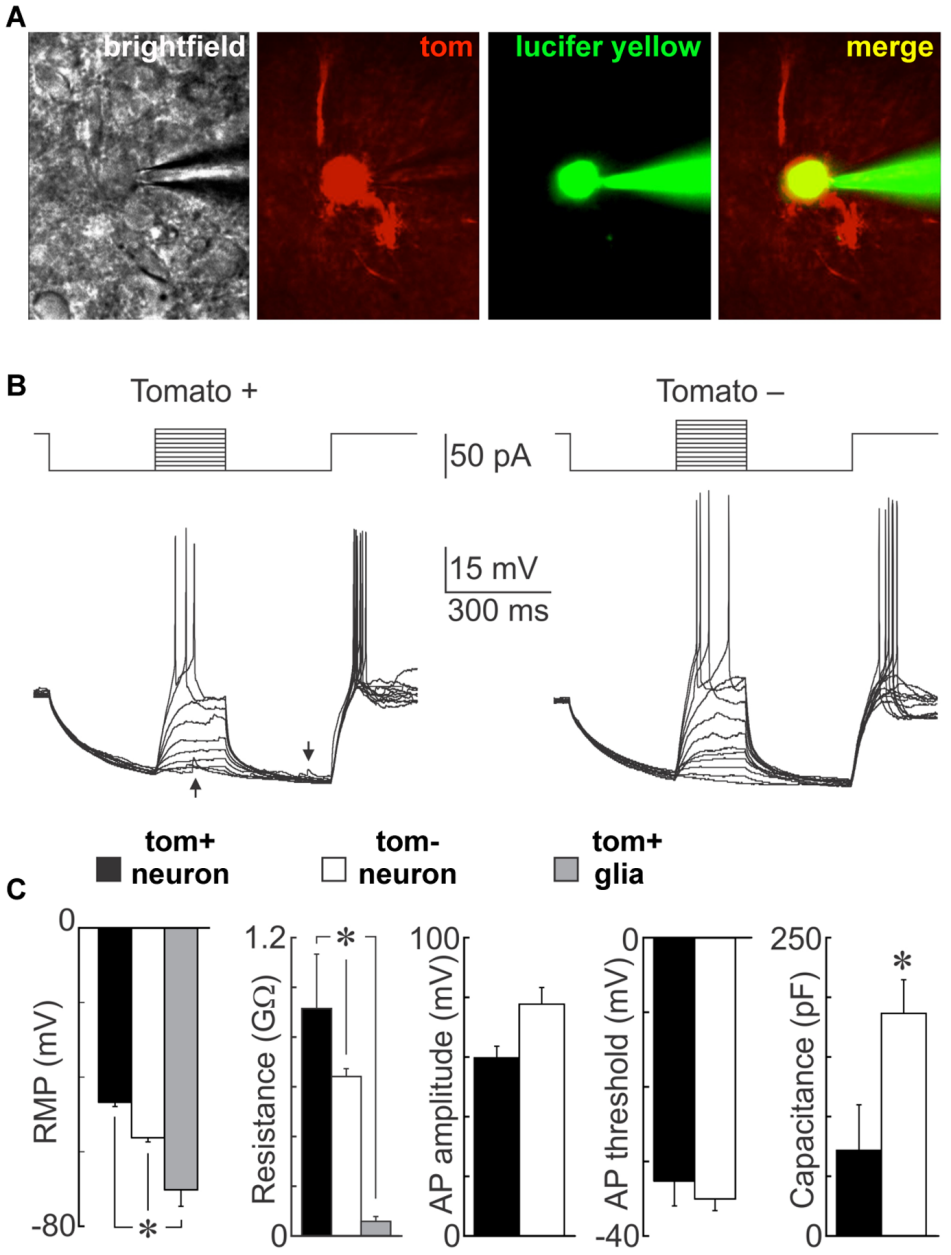
Electrophysiological properties of NG2-glia derived neurons. A neuron targeted by a patch pipette in a living hypothalamic slice is visible by brightfield, expresses Tom, and is filled with Lucifer yellow during the course of recording (**A**). Whole cell voltage recordings obtained from a Tom positive (left) and a Tom negative (right) neuron (**B**). The traces show superimposed voltage responses (lower) to current pulses (upper). Note that both types of cells can fire action potentials. Synaptic potentials are visible in the Tom+ neuron (arrows). Bar graphs compare mean (± SEM) resting and active membrane parameters recorded from excitable Tom+ neurons, conventional neurons (Tom negative neurons) and putative glia (unexcitable Tom+ cells)(n = 4) (**C**). RMP = resting membrane potential, AP = action potential, *p<0.05.

To assess whether NG2-glia produce functional neurons, we performed whole cell current clamp recordings from visually identified Tom+ cells in coronal hypothalamic slices from mice 60 days after TX treatment (Fig. 6A). As illustrated in Figure 6B, a subset of the Tom+ cells were capable of firing overshooting all or none action potentials in response to current injection (n = 4), a feature that unequivocally identifies these NG2-glia derivatives as neurons. Spontaneous post-synaptic potentials were visible (arrows in Fig. 6B), suggesting that the patched cells received synaptic inputs and are therefore integrated within local circuitry. Interestingly, the resting membrane potential (RMP) of Tom-positive neurons (−46.7±1.1 mV) was significantly less hyperpolarised than that observed in neighbouring Tom-negative neurons (−56.3±1.1 mV) or putative Tom-positive glia (−70.2±10.8 mV; Fig. 6C). Input resistances were significantly higher in Tom+ (0.915±0.219 GΩ), versus Tom- neurons (0.641±0.032 GΩ) and putative Tom+ glia (0.070±0.011 GΩ), while the capacitance was substantially lower in Tom+ neurons (71.75±38.12 pF vs. 186.48±28.17 in Tom- neurons; p = 0.04). There was a trend towards lower action potential (AP) amplitudes in Tom+ neurons compared to Tom- neurons, but this did not reach significance. The observed differences are remarkable, as they appear reminiscent of the differences seen between mature dentate gyrus granule cells (DGCs) and adult-born immature DGCs in the hippocampus. Immature DGCs have been shown to exhibit a less hyperpolarised RMP, higher input resistance, lower capacitance, lower amplitude, and lower current threshold when compared to mature DGCs [35]–[37]. Notably, the observed lower capacitance of Tom+ neurons could be seen as indication of a much smaller surface area as compared to Tom- neurons, as would be predicted for recently differentiated cells with less extensive arborisation. Together, these findings suggest that the detected Tom+ neurons have an immature status, which sets them apart from Tom- neurons of the hypothalamus and could be considered as further evidence that Tom+ neurons are adult-born.

## Discussion

### Validity of NG2creER-based Fate Mapping

We present evidence that hypothalamic NG2-glia not only give rise to oligodendrocytes, but also to cells that express neuronal makers and exhibit electrophysiological properties of neurons. The data extends previous studies showing that some neurons in the piriform cortex originate from adult NG2-glia [21], [38]. However, other studies have refuted the notion that adult NG2-glia are neurogenic based on CreER-mediated PDGFRα/NG2 fate mapping [14], [24], [30]. The few sporadic neurons that were fluorescent reporter positive after TX treatment in those studies were interpreted as being due to CreER misexpression in neurons because they were detected shortly after TX treatment and did not accumulate over time. In contrast, Rivers et al. [13] observed reporter positive neurons accumulating over time in the piriform cortex employing a PDGFRα-CreER transgene. None of those neurons were detected shortly after TX treatment by these authors, ruling out that reporter expression is simply due to CreER activity in postmitotic neurons and suggesting that they are indeed derivatives of adult NG2-glia. A very recent study using the latter Cre line now argues that these neurons may not be derivatives of local NG2 glia after all, based on the finding that no reporter+ EdU+ neurons could be detected in the piriform cortex even after almost 100% of the NG2-glia population had incorporated the mitotic marker EdU [23]. Because the reporter-positive piriform cortex neurons nevertheless accumulated over time, the authors suggested that they may originate from yet to be identified reporter-positive cells/progenitors that reside elsewhere in the CNS or that they arrive from elsewhere in the body via the circulation.

In the hypothalamus, we similarly show that the numbers of neuronal marker positive Tom+ cells increase over time and are not detected shortly after TX treatment. The initial absence of neurons was assessed at two days after the initial TX injections because assessment of oligodendrocyte differentiation indicated an increase in the proportion of APC+/Tom+ cells even by the seven day timepoint (two days after the final TX injection), suggesting the potential for very rapid differentiation away from an NG2-glial identity. Given that TX has a serum half-life of only 12 hours in mice [39], [40] and that recombination in NG2-glia is completed by seven days after the first injection, it does not seem possible for the steady accumulation of neurons seen in the period between seven and 60 days to be due to an extended ectopic recombination in postmitotic neurons. That Tom+ neurons are indeed not the result of aberrant recombination in postmitotic neurons is further supported by the detection of Hu+/Tom+/BrdU+ triple labelled cells after delivering BrdU through drinking water. The presence of BrdU indicates that these cells had divided during the BrdU delivery period, and therefore could not have already been postmitotic neurons at the time of Tom induction. This finding is in contrast to the results from a recent study in which an absence of EdU co-labelling led to the conclusion that neurogenesis does not occur in the cortex [23]. The reported differences may reflect a regional variation in the neurogenic capacity of NG2-glia, or result from methodological disparities between the two studies. Our mitotic labelling approach based on 28 days of BrdU in drinking water yielded only about 30% of Tom+ NG2-glia that were also BrdU+. This falls short of the near 100% EdU uptake reported recently [23], [41], but is more compatible with earlier reports employing BrdU [13], [14]. Our failure to label all NG2-glia could be due to the use of different mitotic labels, methodological differences, and/or a labelling period shorter (28 days) than the 36-day duration most recently estimated as the cell cycle length of a cortical NG2-glial cell in a 60 day old mouse [41]. As the same study found variations in the cell cycle length in different grey matter regions, it is possible that the cell cycle of hypothalamic NG2-glia is relatively longer, or that the hypothalamus may even harbour a quiescent NG2-glia population not seen elsewhere. Nevertheless, the degree of BrdU incorporation in Tom+ neurons (∼25%) was in a similar range to the degree of incorporation into the NG2+ population as a whole (∼30%), suggesting that the number of Hu+/Tom+/BrdU+ cells to be found in the adult hypothalamus depends on the efficiency of BrdU+ incorporation. Our finding that not all NG2-glia-derived neurons expressed BrdU may be partially attributable to the very long NG2-glia cell cycle duration. Alternatively, a postmitotic NG2-glia population may exist in the adult hypothalamus, and it may be that recent mitosis is not required for the differentiation of NG2-glia, as self-renewing divisions of adjacent NG2-glia can replace the differentiating cells. Although this idea is somewhat unconventional with respect to neurogenesis, it is supported by recent evidence that NG2-glia undergoing oligodendrogenesis can differentiate directly without proliferating first [42]. Finally, our electrophysiological examination of hypothalamic Tom+ cells suggests an immature neuronal phenotype, strongly arguing against the possibility that Tom+ neurons result from ectopic expression of the NG2-Cre transgene in long-term resident neurons of the adult hypothalamus.

### Sox2 Expression in Hypothalamic NG2-glia

We report that a subset of hypothalamic NG2-glia robustly express the transcription factor Sox2, which has been shown to mark stem cells and is known for its role in pluripotency maintenance and induction [26], [27]. Our finding is noteworthy because hypothalamic Sox2+ cells have been recently shown to give rise to cells with various neural fates in the adult hypothalamus, including neuronal marker expressing cells, using lentivirus-mediated fate tracing [43]. Given that the mitotically active Sox2+ population almost exclusively expresses NG2, it is plausible that the neurogenic/gliogenic Sox2 cells identified by Li et al. [7] are in fact NG2-glia. Interestingly, when we examined Sox2 expression in NG2+ closely apposed cell pairs that likely resulted from a recent cell division, we found pairs with both cells expressing Sox2 at appreciable levels, but also cell doublets in which the Sox2 immunohistochemical signal was absent or barely detectable in both or only one of the cells. Consistent with this, time-lapse monitoring of NG2-glia in acute, postnatal brain slices has revealed both symmetric and asymmetric cell division in NG2-glia [30]. Sox2 expression in NG2-glia may be an indication of mitotic status or multipotent potential. It would therefore be important to conduct time lapse imaging of fluorescent cells in slices from NG2-CreER:Tom mice infected with a Sox2-GFP viral construct, in order to determine whether a downregulation of Sox2 expression is indeed associated with terminal differentiation of daughter cells.

### Stem and/or Progenitor Cells in the Hypothalamus

We show here that NG2-glia account for a high proportion of mitotic cells in the hypothalamic parenchyma. We further find that they both undergo self-renewing divisions in line with a population maintenance function, and that they differentiate into other mature cell types. The comparatively high rate of oligodendrogenesis (25.6% of Tom+ cells at 60 days post TX) implies that, as with other regions of the CNS, the main role of hypothalamic NG2-glia is to provide a continuous source of myelinating oligodendrocytes.

After 60 days, a significant minority (8.6%) of Tom+ cells have adopted a neuronal fate, which is compatible with previous studies in which we and others have shown that both oligodendrocytes and neurons are generated throughout adulthood in the hypothalamus [1], [4], [44]. That these newborn neurons are likely to be functional has previously been inferred from their reaction to stimuli, such as phosphorylation of STAT3 in response to leptin administration [1], [5] or expression of c-fos in response to fasting [5]. We extend this work by demonstrating that newborn hypothalamic cells exhibit the electrical membrane properties of bona fide neurons, fire action potentials and appear to receive input from local circuits. Intriguingly, the deviations between the observed electrical properties of NG2-glia derived neurons vs. Tom- resident neurons appears reminiscent of the differences found between mature and immature dentate gyrus neurons, which are constitutively born in the hippocampal SGZ throughout adult life and thought to be vital to the normal functioning of hippocampal circuits [36], [37], [45]–[47]. In the light of this, it will be intriguing to determine whether this immaturity merely reflects a transient developmental stage that per se has no functional relevance, or whether such immature properties are actively required for proper functioning of the hypothalamic circuitry.

Much of the recent work investigating stem and/or progenitor cells in the hypothalamus has been focused on the role of the tanycytes that line the basal portion of the posterior third ventricle. Lineage tracing to date has not provided any evidence that tanycytes give rise to NG2-glia [5], [9], [10]. Furthermore, mitotic blocker or laser mediated ablation of NG2 glia indicate that adult NG2-glia are generated solely by self-renewal in periventricular brain regions such as the hypothalamus [25], and likely in most other brain regions as well [42]. It thus appears that NG2-glia and tanycytes represent fully separate populations with regard to their adult genesis, yet both have a capacity to act as neuronal precursors, with NG2 cells apparently exhibiting a higher neurogenic potency (this study) compared to tanycytes [9]. It will be interesting to learn whether they nevertheless perform convergent roles in the maintenance of hypothalamic neuronal networks and their functions.

## Materials and Methods

### Ethics

All animal procedures were carried out in accordance with the recommendations of the Canadian Council on Animal Care (CCAC), and have been approved by the McGill University Animal Care Committee.

### Animals

NG2creER^TM^BAC mice [30] were crossed with B6;129S6-*Gt*(*ROSA*)*26Sor^tm9^* ^(*CAG-tdTomato*)*Hze*^/J reporter mice [48] to generate NG2-CreER:tdTomato animals which carry one copy of the Cre transgene and were heterozygous for the Tom reporter allele. Mice were housed in a 12 hr:12 hr light:dark cycle with lights on at 7 am. All experiments were performed on 8 to 12 week old males.

### BrdU Treatment

For icv infusions, osmotic minipumps (flow rate 0.5 µl/hr, 7 day infusion, Alzet) were filled with BrdU (Sigma) dissolved in artificial cerebrospinal fluid (aCSF) resulting in a dose of 30 µg/day. The tubing (Plastics One) connecting the pump to the cannula (Plastics One, cut 2.5 mm below pedestal) was cut to 67 mm, and filled with aCSF. This allowed mice to receive aCSF only for the first two days post-surgery, ensuring that BrdU does not reach the brain until after the recovery period. The cannula was implanted into the right lateral ventricle at stereotaxic coordinates 0.3 mm (anteroposterior), 1.0 mm (lateral), and 2.5 mm (dorsoventral, below skull) as previously described [2]. For intraperitoneal BrdU delivery, a solution of 6.25 µg/ul in saline was injected at a dose of 50 mg/kg body weight twice per day. For oral delivery, BrdU was added to the drinking water at a concentration of 0.8 mg/mL, and supplied *ad libitum*.

### EdU Treatment

The mitotic marker EdU (5-ethynyl–2′-deoxyuridine) was administered via osmotic minipumps (2.5 µg/ µl aCSF) at a dose of 30 µg/day as described for BrdU. Incorporation of EdU into genomic DNA was visualized using a click chemistry kit (Click-it kit, Life Technologies, cat# C10337). Because reaction of the fluorescent azide label with EdU is typically non-saturating, BrdU antibodies may bind to unreacted EdU, causing unwanted cross-reactivity. To prevent cross-reaction, brain sections were treated with the fluorescent azide according to kit instructions followed by incubation with saturating levels (20 mM) of a non-fluorescent azide (O-(2-aminoethyl)-O’-(2-azidoethyl)-pentaethylene glycol, Sigma, cat# 76172), which transforms residual unreacted EdU into a derivative that is not recognized by the BrdU antibody [49].

### Tamoxifen Treatment

Tamoxifen (Sigma) was dissolved in a 1∶9 (v/v) ethanol:sunflower oil mix to a final concentration of 30 mg/mL. Mice were given an intraperitoneal injection with a dose of 1.2 mg TX at 2 hours after lights on and a dose of 1.5 mg at 1 hour before lights off, for 5 consecutive days.

### Tissue Processing

Brain tissue was fixed and processed for immunohistochemical detection as previously described [2]. Briefly, mice were anesthetized with a ketamine (50 mg/kg)/xylazine (5 mg/kg) cocktail, and transcardially perfused with 0.9% NaCL followed by 10% formalin. Brains were removed, postfixed in 10% formalin overnight and then incubated with 20% sucrose in saline for at least 16 hours. 25 µm thick coronal sections were cut using a sliding microtome (Leica, model SM2000R). Sections were collected in 5 series per brain, encompassing the hypothalamic region defined by the anterior-posterior extent of the third ventricle. For immunohistochemistry, sections were first blocked with a solution of 3% goat serum +0.1% triton X-100 in PBS for 1 hour and then incubated with primary antibody in blocking solution at room temperature overnight. Sections containing BrdU underwent an additional antigen retrieval procedure prior to blocking, consisting of either a 10 minute incubation in a 500 U/ml DNase I (Sigma, cat# DN25) solution or a 1 hour incubation in 2N HCl at room temperature. Primary antibodies were used at the following dilutions: rat monoclonal anti-BrdU (Accurate, cat# OBT00030) 1∶500, rabbit polyclonal NG2 (Millipore, cat# AB5320) 1∶250, rat monoclonal CD13 (BD Pharminogen, cat# 558744) 1∶200, mouse monoclonal NeuN (Millipore, cat# MAB377) 1∶200, mouse monoclonal HuC/D (Life Technologies, cat# A21271) 1∶20, mouse monoclonal APC (EMD, cat# OP80) 1∶100, mouse monoclonal GFAP (Millipore, cat# MAB360) 1∶500, mouse monoclonal Sox2 (R&D Systems, cat# MAB2018) 1∶100. In some sections, Tomato fluorescence was enhanced by labelling with an anti-RFP rabbit polyclonal antibody (Rockland Immunochemicals, cat# 600-401-379), 1∶3000. Alexa350, Alexa488, Alexa568 and Alexa647 conjugates (Life Technologies) were employed as secondary antibodies for fluorescence detection.

### Data Analysis and Cell Counts

Epifluorescent images of brain sections were taken using a Zeiss Axio Image D2 microscope equipped with an AxioCam HRm camera. For quantification of cells, images were taken using a 10× objective of every fifth coronal section between the anterior edge of the median eminence (ME, Bregma –1.34 mm) and the posterior boundary of the third ventricle (Bregma –2.54 mm) [50]. Images were positioned with the midline of the third ventricle to one side and the lower right or left corner of the frame coincident with the ventral aspect of the median eminence, giving a counting frame size of 1.0 mm (medio-lateral) ×1.4 mm (ventro-dorsal), covering most of one hemispheric portion of the hypothalamus. Cell counts included all fully visible cells within the counting frame.

For neural marker expression analysis, confocal images were acquired using the visible laser scanning and spectral descan detection portion of an Olympus FV1000MPE Basic system (Olympus) equipped with a ZDeck motorized stage (Prior Scientific Inc.). Low magnification images were collected using a 10× objective (UPLNSAPO, Olympus) at a resolution of 1600×1600 pixels, and high magnification images were obtained using a 60× oil objective (PLAPON, Olympus) at a resolution of 1024×1024. Z-stacks were acquired with a 50% overlap between adjacent 0.9 µm thick optical slices. The axial range was set to cover the entire thickness of the brain slice (25–30 um, 55–70 images). A maximum intensity projection (MIP) of the z-stacks was then created using the Olympus Fluoview software.

### Whole Cell Recordings

Patch micropipettes were prepared by pulling glass capillary tubes (1.2 mm o.d., A-M Systems Inc.) on a micropipette puller (P-87; Sutter Instrument, Novato, CA). Micropipettes were filled with a solution containing 140 mM potassium gluconate, 2 mM MgCl_2_, 10 mM HEPES, 2 mM Na_2_ATP and 0.4 mM NaGTP (pH adjusted to 7.25 with NaOH), to which 0.5% Lucifer Yellow (Molecular Probes, Life Technologies, Invitrogen) was added. Pipette resistance in the bath was 3 to 5 MΩ. Cells were visualized using a 60X water immersion objective attached to an Olympus BX51WI upright microscope equipped with a digital camera (Coolsnap cf2, Photometrics Inc., Tucson, AZ) and desktop computer running RS Image software (Roper Scientific; Ottobrunn, Germany). Electrodes were visually guided to target cells using a motorized micromanipulator (SD Instruments Inc., Grants Pass, OR). Positive pipette pressure was maintained while the electrode was directed towards the target cell in order to minimize debris influx, and sweeping the pressurized flow over the target cell appears to increase the likelihood that a clean giga-ohm seal is made prior to whole cell recordings. Under these conditions, pipette series resistance was 10 to 30 MΩ.

Whole cell current and voltage (d.c. 2 KHz) was recorded using a multiclamp amplifier (Axon 700B, Molecular Devices) attached to a Digidata 1440A interface and digitized using pClamp 10. Signals were analyzed offline using Clampfit 10 software.

### Statistics

All graphs show mean ± SEM. With regard to cell counts, results were averaged from at least 4 sections per animal, and 3–6 animals were analysed per condition. Significant differences between groups of data were calculated using the Student’s *t*-test.; ns = non significant (p>0.05), *p≤0.05, **p<0.005.

## Supporting Information

Figure S1 **Fidelity of the NG2creER:tdTomato reporter.** Mice were treated with TX twice daily for two days. (**A**) Cell types were determined by colocalisation of Tom with antibodies to NG2 to identify NG2-glia (arrowhead N), CD13 to identify pericytes (arrowhead P) and APC to identify oligodendrocytes (arrowhead O). (**B**) Quantification of results from **A**. Labelling with antibodies to HuC/D (**C**) and NeuN (**D**) confirmed that Tom+ neurons were not present at this timepoint.
(TIF)
