# Supplementary figures and images for: Evidence for NG2-glia Derived, Adult-Born Functional Neurons in the Hypothalamus
Source: PLoS One. 2013 Oct 29;8(10):e78236. doi: 10.1371/journal.pone.0078236 (PMC3812154; doi:10.1371/journal.pone.0078236)

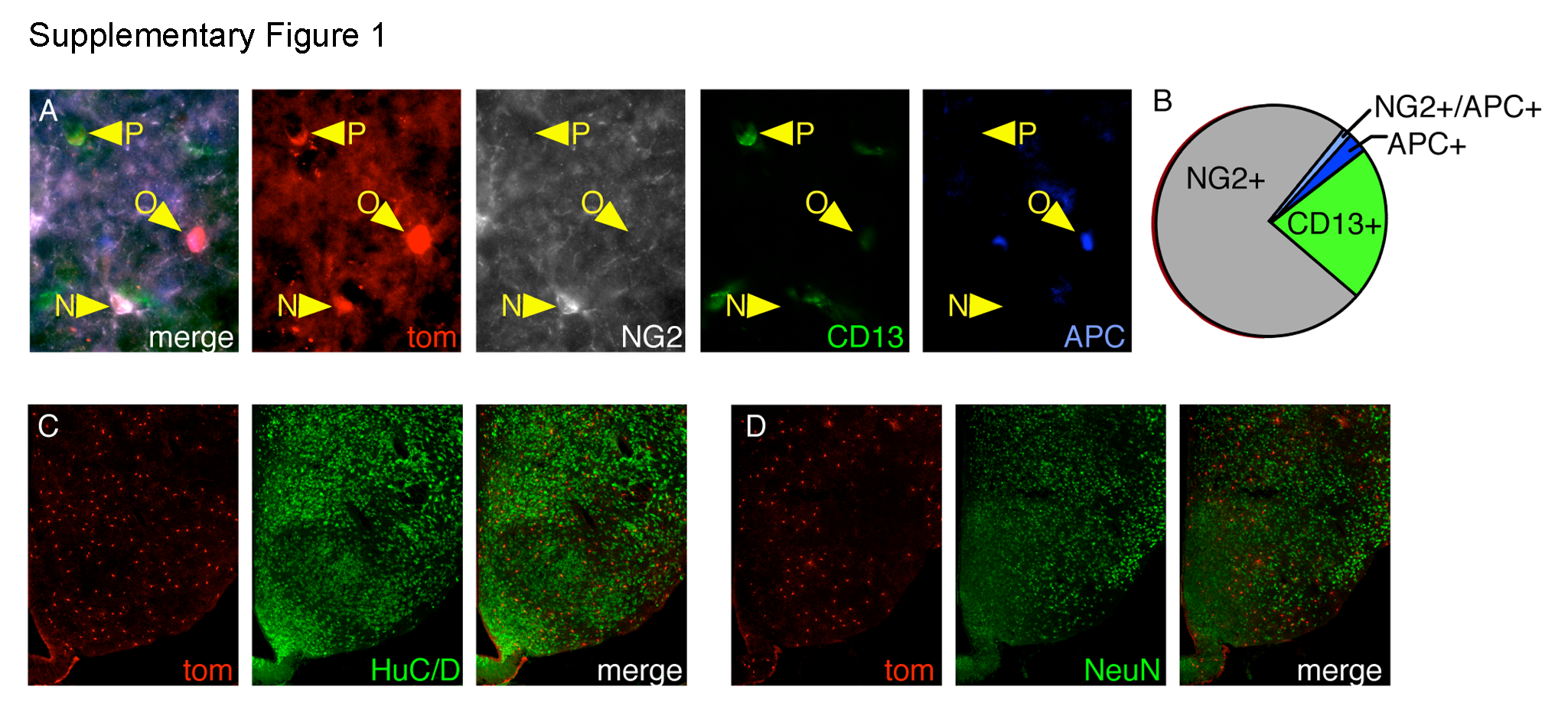

Supplement: Figure S1 — Fidelity of the NG2creER:tdTomato reporter. Mice were treated with TX twice daily for two days. (A) Cell types were determined by colocalisation of Tom with antibodies to NG2 to identify NG2-glia (arrowhead N), CD13 to identify pericytes (arrowhead P) and APC to identify oligodendrocytes (arrowhead O). (B) Quantification of results from A. Labelling with antibodies to HuC/D (C) and NeuN (D) confirmed that Tom+ neurons were not present at this timepoint. (TIF) [file pone.0078236.s001.tif]
